# Regulating the stem cell industry: needs and responsibilities

**DOI:** 10.2471/BLT.16.189977

**Published:** 2017-07-01

**Authors:** Tsung-Ling Lee, Tamra Lysaght, Wendy Lipworth, Tereza Hendl, Ian Kerridge, Megan Munsie, Cameron Stewart

**Affiliations:** a Centre for Biomedical Ethics, Clinical Research Centre, National University of Singapore, #02-03, 10 Medical Drive, 117597 Singapore.; b Centre for Values, Ethics and the Law in Medicine, Sydney, University of Sydney, Australia.; c Stem Cells Australia, University of Melbourne, Melbourne, Australia.; d Sydney Law School, University of Sydney, Sydney, Australia.

Emerging biotechnologies pose public health challenges^1^ because of both the known and unforeseen risks they carry, the uncertain medical benefits they offer, the speed at which they have been disseminated and their unproven mode of application.^2^ The development of therapies from advances in stem cell science reveals the need to pay critical attention to stem cell treatments. Stem cells have attracted scientific, clinical and public interest because they are self-renewing and have the capacity to develop into specific cell types, depending on the source of stem cells and their biological plasticity. The hope is that stem cells could be used either to replace damaged cells or to create an environment for cellular regeneration to treat several conditions, including osteoarthritis, diabetes, macular degeneration and Parkinson disease.

Although promising in theory, so far very few stem cell therapies have proven to be safe and effective in clinical trials. Yet, despite the absence of evidence to support their use, there has been a global proliferation of clinics and associated businesses offering stem cell-based interventions to patients having serious medical conditions.^3^ These clinics operate mostly in the private health-care sector and typically market their interventions directly to patients over the internet. The emergence of these clinics has not only created domestic markets in many high-income countries,^3^ but has also fomented stem cell tourism – the movement of people across international boundaries to access putative stem cell treatments. The global reach of this expanding industry exploits weaknesses and differences in national regulatory infrastructures^4^ and has revealed the need for an international approach to report and monitor the harms and benefits of these putative treatments.

Although once limited to low-to-middle income countries with weak regulatory infrastructure, providers of unproven stem cell therapies are operating in high-income countries that have sophisticated biomedical regulatory systems.^5^ In these otherwise highly regulated jurisdictions, providers of such therapies operate in an under-regulated domain – the grey zone between clinical practice, research and innovation. In some cases, these grey zones emerged because regulators introduced risk-based frameworks that excluded or exempted certain uses of stem cells from the mechanisms that regulate other biological and non-biological therapies. Regulators also accepted stem cell interventions as part of the standard practice of medicine.

These regulatory weaknesses constitute a public health issue in at least three ways. First, many patients may be harmed by unproven stem cell therapies. While patients have the right to choose or refuse medical therapies that may carry significant risk, the validity of these choices depends on the information they have been given about the therapies and on their capacity to make informed choices. However, patients who seek stem cell interventions are often vulnerable and may be desperate for any treatment that they believe will save their life or improve its quality. They may also assume that such therapies are safe and effective, and trust not only that their medical providers are competent and have their best interests at heart, but also that these stem cell therapies are appropriately regulated.

Second, because the industry is poorly regulated, it is subject to little public scrutiny and accountability. This lack of transparency enables clinicians to offer unproven and potentially unsafe therapies and to set their fees according to whatever the private health-care market will bear, without any form of regulatory or medical control. The interventions offered by stem cell providers can cost from 5000 United States Dollars (US$) to over US$ 100 000, not including travel expenses or follow-up care.^6^ For many patients unable to afford the high service fees, funding is sought through debt financing (e.g. mortgaging their house) or community and charity fundraising, among others. This may make patients financially vulnerable, have significant psychosocial consequences and deprive individuals and communities of resources that could be spent elsewhere. Additionally, in some countries, the costs of adverse health effects caused by stem cell interventions are borne by public health-care systems, not by stem cell clinics or patients.

Third, regulatory failure not only enables unscrupulous providers to operate with little oversight, but also means that adverse effects are likely to be under-reported, as patients who are harmed by failed stem cell interventions rarely seek legal redress. Because of this lack of oversight, patients harmed by these interventions – and their families – may also be affected financially; as well, objective data on the impact of this industry on patients and on public health systems may be incomplete.

Therefore, failure to effectively regulate the stem cell industry may have a range of detrimental effects on patients, their families, public health systems, research and public trust in stem cell science and biomedical science in general. Given the local and global significance of these threats, it is important to consider the role of global organizations, particularly the World Health Organization (WHO), in regulating and containing the stem cell industry.

The ethical, social and public concerns raised by stem cell interventions have prompted the International Society of Stem Cell Research (ISSCR), an international non-profit organization of stem cell scientists, to issue the *Guidelines for stem cell research and clinical translation*.^7^ While such voluntary guidelines are useful, they lack the political, legal and moral authority that guidelines from WHO may offer. Furthermore, if WHO were to adopt stem cell product regulations under Article 21 of its Constitution, all Member States would be required to take the corresponding legislative steps unless they expressed reservations.^8^ This move would also help to strengthen national regulatory landscapes^9^ and assist sovereign governments to face potential political opposition to such regulation.^10^

While some may question whether it is appropriate for WHO to engage in what might seem a highly specific scientific and clinical concern, Article 4 of the United Nations Education, Scientific and Cultural Organization (UNESCO) *Universal Declaration on Bioethics and Human Rights*^10^ recognizes that “In applying and advancing scientific knowledge, medical practice and associated technologies, human vulnerability should be taken into account. Individuals and groups of special vulnerability should be protected and the personal integrity of such individuals respected.”^11^

The Declaration does not impose a positive duty on governments to mitigate every kind of human vulnerability. It does, however, emphasize the need for governments and the global community to be aware of the contexts where vulnerability arises and could be exploited, and to take measures to mitigate such exploitation. Article 14 of the Declaration also provides that “The promotion of health and social development for their people is a critical purpose of government that all sectors of society share.” In regulatory context, this means that national governments should regulate biomedical research and prevent fraud; this should be coupled with a coherent international response to promote the regulation of the clinical application, global production, sales and marketing of proven and unproven stem cell therapies.

There is precedent for such action because WHO has previously addressed regulatory, governance and health issues associated with other health industries that run parallel to, or counter, established health systems and clinical practices. For example, following WHO *Guidelines on developing consumer information on proper use of traditional, complementary and alternative medicines*,^12^ some Member States have chosen to regulate practices and products of traditional, alternative and complementary medicines.

If the adoption of regulations under Article 21 proved politically challenging, WHO could instead develop a code of practice drawn from the ISSCR guidelines. This would encourage sharing and gathering of evidence on safety and efficacy before the commercial provision of stem cells, and clarify the ethical principles that should underpin national laws and regulations regarding clinical practice.

Other possible roles for WHO might be to: provide much-needed technical guidance to resource-poor countries; use its mechanisms to gather and disseminate expert advice; convene expert advisory panels and committees on issues regarding the manufacture, licensing, regulation and proper use of stem cells; provide a platform for cross-jurisdiction information sharing; and establish a global governance framework for monitoring countries’ progress in regulating the stem cell industry. Such a platform may encourage cross-country learning and help identifying and aligning best practices in the standards of care across jurisdictions.

To tackle the issues associated to the national and international under-regulation of the stem cell industry, a global strategy needs to be put in place. This strategy, which could be developed by WHO, should moderate the global stem cell industry, protect global health and public safety and promote future research to increase the evidence base of the stem cell industry.
